# Effect of an in-consult patient decision aid on decisional quality and involvement for patients with severe hip or knee osteoarthritis: a multicenter, cluster randomized controlled trial

**DOI:** 10.2340/17453674.2026.45697

**Published:** 2026-04-08

**Authors:** Trine Ahlmann PEDERSEN, Martin LINDBERG-LARSEN, Charlotte Myhre JENSEN, Karina Dahl STEFFENSEN, Claus VARNUM

**Affiliations:** 1 Department of Orthopedic Surgery, Lillebaelt Hospital – Vejle, University Hospital of Southern Denmark, Vejle; 2 Department of Regional Health Research, University of Southern Denmark; 3 Department of Orthopedic Surgery and Traumatology, Odense University Hospital, Odense; 4 Orthopedic Research Unit, Department of Clinical Research, University of Southern Denmark; 5 Center for Shared Decision Making, Lillebaelt Hospital – Vejle, University Hospital of Southern Denmark; 6 Faculty of Medicine, Aalborg University, Aalborg, Denmark

## Abstract

**Background and purpose:**

Shared decision-making (SDM) is important in managing patients with severe hip and knee osteoarthritis, where treatment decisions often depend on patient preferences and values. We aimed to evaluate the effectiveness of an in-consult patient decision aid in improving decision quality and patient involvement compared with usual care.

**Methods:**

In a cluster-randomized controlled trial, surgeons from 2 Danish orthopedic departments were randomized to use in-consult patient decision aids or provide usual care. The primary outcome was decision quality assessed using the Hip/Knee OA Decision Quality Instrument (HK-DQI). Patients who were well informed and received their preferred treatment received an Informed Patient-Centered (IPC) score of 1, indicating good decisional quality; all others scored 0. Secondary outcomes included patient-experienced engagement and involvement in decision-making, assessed using CollaboRATE and the decision-process domain of the Hip/Knee OA Decision Quality Instrument and consultation duration. Relative risks (RR) and mean differences (MD) with 95% confidence intervals (CIs) were estimated using multilevel regression models.

**Results:**

20 surgeons were randomized to either use in-consult patient decision aids (n = 10) or provide usual care (n = 10). Of the 1,038 patients screened for severe hip or knee osteoarthritis, 581 were included in the study. Decision quality was similar between groups, with a score of 43 (36–51) in the intervention group and 44 (37–52) in the usual care group (RR 0.9, CI 0.7–1.2; P = 0.8), likewise, patient-experienced engagement assessed with CollaboRATE (RR 1.1, CI 0.8–1.4; P = 0.6). Consultations were longer in the intervention group, with a mean difference of 3.9 minutes (CI 0.1–7.7; P = 0.04).

**Conclusion:**

Decision quality and patient-reported engagement were similar between groups, while consultation time was longer with in-consult patient decision aids use. In the absence of prespecified minimal important differences, the clinical relevance of these findings remains uncertain.

Although joint replacement—including total hip replacement (THR), total knee replacement (TKR), or unicompartmental knee replacement (UKR)—has shown effectiveness in reducing pain, improving function, and enhancing health-related quality of life, dissatisfaction persist with rates of approximately 7% for THR and about 18% and 12% for UKR and TKR, respectively [1,2].

Shared decision-making (SDM) is recommended for preference-sensitive decisions, such as choosing the treatment option—whether surgical or non-surgical—that best aligns with the patient’s values and preferences among the available alternatives for hip and knee osteoarthritis (OA) [3]. In-consult patient decision aids assist this process by helping surgeons clarify patients’ goals and concerns, inform patients about treatment options, and outline the advantages and disadvantages of each treatment [4]. Patients who actively participate in decision-making tend to build stronger relationships with their surgeons, develop more realistic treatment expectations, and are more likely to receive care aligned with their preferences [5]. The specific impact of SDM on joint replacement remains unclear, which is why a gap persists in knowing whether SDM enhances decision quality, satisfaction, or health outcomes for patients with hip or knee OA [5].

This Danish multicenter cluster-randomized controlled trial evaluated whether SDM and using an in-consult patient decision aid influenced decision quality, patient engagement, involvement, and length of the consultation compared with standard practice involving patients with severe hip or knee OA who were referred to hospital for treatment.

## Methods

In this multicenter, cluster-randomized, controlled superiority trial, orthopedic surgeons (clusters) specializing in joint replacement of the hip or knee were randomly assigned to perform SDM supported by an in-consult patient decision aid (intervention) or to continue usual consultations without an in-consult patient decision aid (control) in a 1:1 ratio stratified by hospital. The study adheres to the Consolidated Standards of Reporting Trials (CONSORT) extended guidelines for C-RCTs [6] and the Standards for Universal Reporting of Decision Aids Evaluation (SUNDAE) [7]. The detailed trial protocol has been published [8]. Primary, secondary, and exploratory outcomes were assessed at 1 week post-consultation. Explorative outcomes, including clinical and health-related follow-up data for 3 and 12 months, will be reported separately in a forthcoming follow-up study [8].

### Participants

Eligible participants were individuals aged 18 years or older, diagnosed with severe primary OA, and considered suitable candidates for THR, TKR, or UKR. Additional criteria included the ability to read and understand Danish, the capacity to provide informed consent, and access to E-Boks, the official Danish digital mailbox. Patients with prior joint replacement, non-OA diagnoses, or cognitive impairments were excluded [8]. During October 2023 to November 2024, participants were recruited from orthopedic outpatient clinics at 2 university hospitals in Denmark.

### Interventions

Before conducting this trial, 2 in-consult patient decision aids were developed and evaluated for individuals with severe hip and knee osteoarthritis. Both aids, entitled “Which Treatment Is Right for Me?”, were produced in booklet format and represent the first in-consult patient decision aids for hip and knee osteoarthritis in Denmark [8]. Development was informed by focus group interviews with patients and orthopedic surgeons and carried out in collaboration with clinical stakeholders to ensure relevance and usability.

The aids were subsequently alpha tested to assess feasibility, acceptability, and integration into clinical workflows. Face-to-face interviews followed the approach described by Stacey et al. [9] using the Preparation for Decision-Making Scale (PDMS) [10]. The final versions of the patient decision aids are structured into 5 sections and include option cards detailing the advantages and disadvantages of treatment options (surgical vs non-surgical), simplified statistics (e.g., prosthesis survival rates, surgical risks, and anticipated surgical vs non-surgical outcomes), and patient stories. The development process and results of the alpha testing will be reported separately.

Surgeons in the intervention group attended a 3-hour training session in SDM and the use of the in-consult patient decision aids. This training, scheduled the day before the trial commenced at each of the 2 participating centers, also covered screening, recruitment, intervention implementation, and survey distribution timelines. In addition, surgeons were instructed to use the patient decision aids actively during the consultation in line with the “three-talk model,” a widely recognized framework for practicing SDM [11]. The model structures the conversation into 3 iterative steps: (I) team talk—establishing partnership and emphasizing the patient’s role in decision-making; (II) option talk—presenting and discussing the available options and their risks and benefits; and (III) decision talk—supporting the patient in reflecting on preferences before making a decision. The patient decision aids literature was not intended to be read independently by the patient, but to guide dialogue and discussions in real time. Surgeons decided themselves when to introduce and discuss the individual option cards in relation to other diagnostic assessments, such as the physical mobility test and interpretation of radiographs.

Surgeons continuing usual consultations (controls) attended a mandatory introductory session that focused on trial design, screening tasks, and recruitment procedures.

### Outcome

#### Primary outcome

The primary outcome was patients’ decisional quality after engaging in SDM facilitated by in-consult patient decision aids compared with standard care. Decisional quality was assessed 1 week post-consultation using the Danish-translated and psychometrically tested Hip/Knee OA Decision Quality Instrument (HK-DQI) [8]. The Hip/Knee OA Decision Quality Instrument (HK-DQI) includes:

- *Knowledge score:* Based on 5 items, e.g., “If 100 people undergo hip replacement surgery, how many will experience less hip pain?” (answer options: 30, 50, 70, or 90). Scores range from 0–100%, with >60% classified as “well informed” [12].
- *Preference item:* Assesses treatment preference (surgical, non-surgical, or unsure).

Treatment preferences were compared with treatment received to evaluate concordance (i.e., surgery preference matched with surgery received or non-surgical preference matched with non-surgical treatment). Patients unsure of their preference were categorized as mismatched [13].

Patients who were well informed and had concordant decisions received an Informed Patient-Centered (IPC) score of 1, indicating good decisional quality; all others scored 0. Non-IPC decisions were categorized as: (i) not well informed but concordant, (ii) well informed but non-concordant, or (iii) not well informed and non-concordant [12].

#### Secondary outcomes

Secondary outcomes included patient-experienced engagement and involvement, assessed 1 week post-consultation using CollaboRATE and the Hip/Knee OA Decision Quality Instrument (HK-DQI) SDM process score and the and length of the consultation compared with standard practice measured by time The time duration was documented by the surgeons.

- *CollaboRATE:* A 3-item, patient-reported measure of SDM in clinical encounters, translated and validated in Danish [14]. Patients rated their experience of SDM on a scale from 0 (no effort) to 9 (maximum effort), e.g., “How much effort was made to help you understand your health issues?” To address ceiling effects, a dichotomized “top score” was applied: participants scored 1 if all items were rated 9, otherwise 0 [15].
- *HK-DQI SDM process score:* Assesses patients’ experiences of SDM during consultations, including clinician and patient contributions, e.g., “How much did you and your healthcare providers talk about the reasons you might not want to have hip replacement surgery?” It is based on 5 items scored 0–5, converted to a 0–100% scale, with higher scores reflecting greater SDM(12).

*Consultation length* was recorded based on surgeon-reported time immediately after the consultation.

*Patients’ received treatment* was obtained from medical records 6 months after the consultation.

#### Exploratory outcome

As exploratory outcomes, responses to the Hip/Knee OA Decision Quality Instrument (HK-DQI) value clarification item (“*What matters most to you?*”) were examined descriptively, with particular focus on group-level differences in the importance placed on avoiding surgery.

Differences in planned and received treatment (surgical vs non-surgical) at enrolment and at 6-month follow-up were also explored. In addition, knowledge scores, treatment concordance, and the proportion of patients classified as well informed were assessed descriptively.

Patients’ self-rated health status was measured at baseline using the EQ-5D Visual Analogue Scale (VAS), ranging from 0 (worst imaginable health) to 100 (best imaginable health) [16].

### Deviations from the protocol

The enrolment period deviated by 1 month from the originally estimated 12 months, extending from October 2023 to November 2024. This extension was due to fluctuations in surgeons’ availability caused by a higher turnover of staff during the trial period.

Additionally, while the protocol initially proposed exploring surgeons’ learning curves through descriptive analysis, this was modified. A multilevel linear model with surgeons included as random effects was used to examine the development of consultation lengths in both exposure groups to assess the influence of experience. Consultation length was analyzed using multilevel linear regression rather than the t-test specified in the SAP, as this approach is more appropriate (see Table 2).

Finally, an exploratory-outcomes section was added post hoc to provide a descriptive overview of selected treatment patterns and value-related responses not pre-specified in the original protocol.

### Statistics

A detailed statistical analysis plan (SAP) was made publicly available (NCT05972525) before the enrolment of the last patient. Revisions to the statistical analysis plan were made public before analyses began. An independent statistician performed the analyses. The trial steering group followed published procedures for blinded interpretation of the intention-to-treat analysis. Research staff were not blinded; however, the statistician was blinded. No per-protocol analysis was conducted, as there was no crossover between groups.

Demographic and descriptive data were summarized as count (%) for categorical variables, and as mean with standard deviation (SD) or median with interquartile range (IQR) for continuous variables. Normal distribution was checked using histograms. Binary and continuous outcomes were modeled using multilevel logistic and linear regression, respectively, with surgeons included as random effects to account for cluster randomization. Following model fitting, we estimated marginal risks (MR) and relative risks (MRR) for binary outcomes, and marginal means (MM) and mean differences (MMD) for continuous outcomes, all with 95% confidence intervals (CIs).

2 sensitivity analyses were performed to explore the potential influence of ceiling effect in CollaboRATE scores [17]: a multilevel logistic model of top scores and a multilevel Tobit regression, both with surgeons as random effects [18].

To assess the learning curve for the intervention and control group, consultation length for each surgeon’s first 10 patients and subsequent groups of 10 patients (1–10, 11–20, 21–30, 31–40, 41–50, and 51–60) were analyzed using a multilevel linear model with surgeons as random effects and visualized as MM.

Due to less than 5% missing data, only complete case analyses were performed. A P value < 0.05 was considered statistically significant. The statistician used Stata version 18.0 (StataCorp, College Station, TX, USA) for the analyses.

### Ethics, funding, data sharing, use of AI tools, and disclosures

All patients gave oral and written informed consent. The trial was approved by the General Data Protection Regulation office of the Region of Southern Denmark (Journal No. 22/9955). Ethics committee approval was not required, as the study involved questionnaires only (Ethics application project ID: S-20200137). The trial was registered on ClinicalTrials.gov (NCT05972525, registered August 6, 2023). Data was stored securely in OPEN at Odense University Hospital in compliance with the European General Data Protection Regulation. The study received unrestricted internal funding from the Departments of Orthopedic Surgery at Lillebaelt Hospital–Vejle and Odense University Hospital. External funding came from the Region of Southern Denmark (22/26219) and the Research Fund at Lillebaelt Hospital. Artificial intelligence (AI) tools (ChatGPT, OpenAI) were used solely for language editing and spelling correction. No AI tools were used for data analysis, interpretation of results, or generation of scientific content. The authors take full responsibility for the content of the manuscript. CV received institutional travel expenses from Stryker unrelated to this study; TAP, MLL, CMJ, and KDS declare no competing interests. Complete disclosure of interest forms according to ICMJE are available on the article page, doi: 10.2340/17453674.2026.45697

## Results

### Characteristics of surgeons and patients

**Figure 1:**
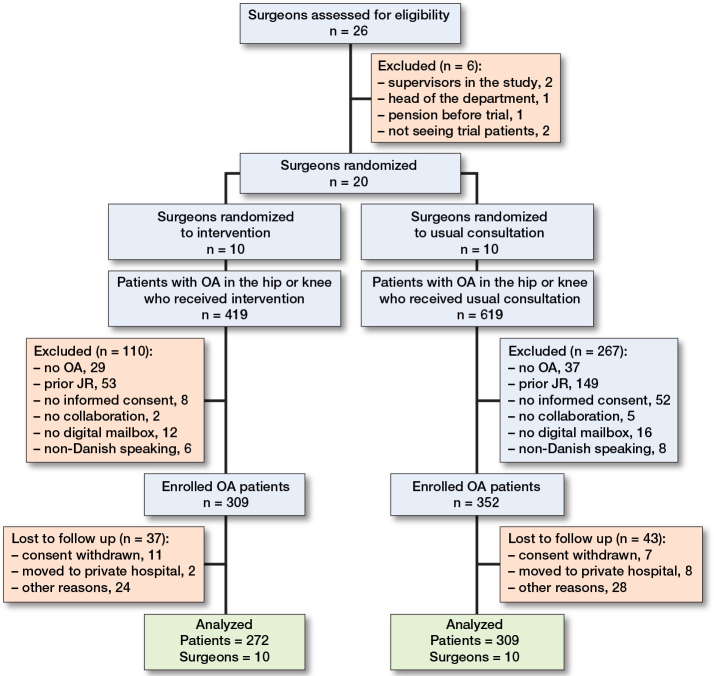
Flowchart showing surgeon and patient inclusion and exclusion for the analytic data set. OA: osteoarthritis, JR: joint replacement.

During the inclusion period, 26 surgeons specializing in hip or knee joint replacement were assessed for eligibility. 6 surgeons were excluded: 2 were trial supervisors, 1 was the head of the department, 1 retired before the trial began, and 2 did not consult relevant trial patients. Hence, 20 surgeons were randomized to either SDM using an in-consult patient decision aid or controls, resulting in 10 surgeons in each group (Figure 1).

During the trial, 4 surgeons left employment; however, they kept their cluster number and were included in the analysis (3 retired, and 1 went on maternity leave). 2 surgeons transferred between centers but retained their randomization group allocation, and 3 new surgeons were employed, enrolled, and received mandatory tasks/training within either the intervention or usual consultation. The median number of patients per surgeon was 29 (range 5–56).

**Figure 2:**
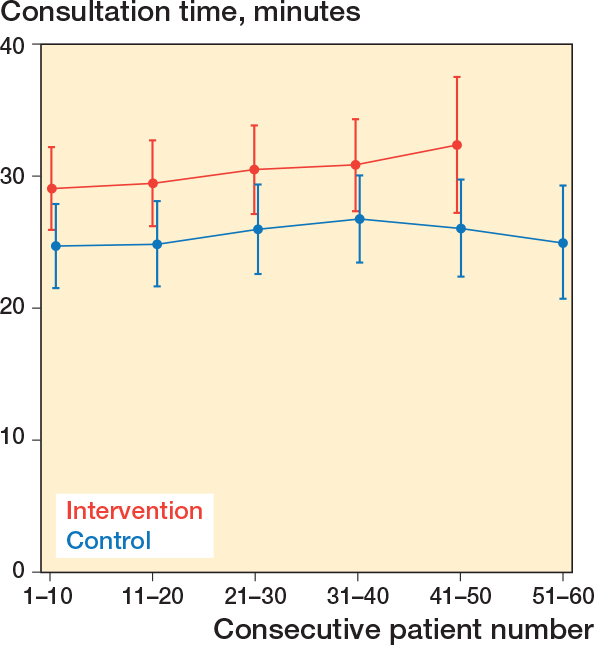
Time consumption with an in-consult patient decision aid (intervention) vs control group, categorized by number of patients enrolled in the trial. Whiskers indicate 95% confidence intervals.

1,038 patients were screened for eligibility of whom 661 (64%) were found to be eligible: 309 patients received the intervention, and 352 were controls. 80 patients were excluded from the analysis due to loss to follow-up, including 18 who withdrew consent, 10 who moved to private hospitals and subsequently withdrew consent, and 52 for various other reasons. Hence, 272 in the intervention group and 309 in the control group were included in the final analyses (Figure 2).

The mean age of the patients was 67 years, 54% were women, and 57% had knee OA. Patient characteristics were generally comparable between the groups with 2 exceptions: first, a higher proportion of patients were scheduled for surgery in the control group (261 patients, 84%) than in the intervention group (170 patients, 62%). Further, among patients with knee OA, the median EQ-VAS score was higher in the intervention group (65, IQR 41–77) than in the control group (50, IQR 40–74) and also higher than the median score for patients with hip OA in the intervention group (50, IQR 40–71) and in the control group (50, IQR 36–72) (Table 1).

**Table 1:** Characteristics of patients and surgeons. Values are count (%) unless otherwise specified

	All	Consultation with an in-consult patient decision aid	Usual consultation
Patients	581	272	309
Age, median (IQR)	67 (59–74)	67 (59–74)	67 (59–74)
Sex			
Women	315 (54)	151 (56)	164 (53)
Men	266 (46)	121 (44)	145 (47)
Joint			
Hip	251 (43)	110 (40)	141 (46)
Knee	330 (57)	162 (60)	168 (54)
Income status			
Salary	205 (35)	92 (34)	113 (37)
Pension etc.	350 (60)	169 (62)	181 (59)
Other	26 (4.5)	11 (4.0)	15 (4.9)
Education			
High school or less	277 (48)	129 (47)	148 (48)
Some college	243 (42)	116 (43)	127 (41)
Graduated college	35 (6.0)	11 (4.0)	24 (7.8)
Other	26 (4.5)	16 (5.9)	10 (3.2)
Civil status			
Living with a partner	427 (73)	197 (72)	230 (74)
Living alone	148 (25)	72 (26)	76 (25)
Other	6 (1.0)	3 (1.1)	3 (1.0)
Treatment scheduled **^a^**			
Surgical	431 (74)	170 (62)	261 (84)
Non-surgical	150 (26)	102 (38)	48 (16)
EQ VAS, median (IQR)			
Hip patients	50 (37–72)	50 (40–71)	50 (36–72)
Knee patients	60 (41–77)	65 (41–77)	50 (40–74)
Surgeons	20	10	10
Sex			
Women	2	1	1
Men	18	9	9
Joint specialty			
Hip	9	5	4
Knee	11	5	6
Working position			
Specialty doctor	9	4	5
Consultant	11	6	5
Site			
Vejle	13	7	6
Odense	7	3	4

IQR: interquartile range.

a Scheduled treatment at enrolment.

### Primary outcome

Good decision quality, defined as achieving an IPC score of 1, was observed in 117 patients in the intervention group, with a score of 43 (36–51), and in 138 patients in the control group, with a score of 44 (37–52) (RR 0.9, CI 0.7–1.2; P = 0.8) (Table 2).

**Table 2:** Results of patients’ IPC decision, involvement, and consultation length

Item	Consultation with an in-consult patient decision aid (n = 272)	Usual consultation (n = 309)	MMD/MRR (CI)	P value
Primary outcome: Informed Patient-Centered decision (IPC) score, MR in % (CI)				
IPC	43 (36–51)	44 (37–52)	0.9 (0.7 to 1.2)	0.8
No IPC score	57 (49–64)	55 (48–62)	1.0 (0.8 to 1.2)	0.7
Not well informed and concordance	27 (21–34)	29 (22–35)	0.9 (0.6 to 1.3)	0.7
Well informed and no concordance	15 (10–20)	14 (9–18)	1.1 (0.6 to 1.6)	0.6
Not well informed and no concordance	15 (8–21)	12 (6–18)	1.2 (0.5 to 1.9)	0.002
Secondary outcome				
CollaboRATE Top score, MR in % (CI)	35 (27–42)	32 (25–39)	1.1 (0.8 to 1.4)	0.6
HK-DQI, SDM process score, MM (CI)	63.3 (59.9–66.8)	60.1 (56.7–63.5)	3.3 (–1.5 to 8.0)	0.2
Consultation length, minutes, MM (CI)	29.4 (26.3–32.5)	25.5 (22.4–28.5)	3.9 (0.1 to 7.7)	0.04
Exploratory outcome. HK-DQI: *What matters most to you?*, MM (CI)				
Relieve pain	9.4 (9.2–9.6)	9.3 (9.2–9.5)	0.1 (–0.2 to 0.3)	0.6
Not be limited	9.4 (9.2–9.6)	9.4 (9.2–9.5)	0.0 (–0.2 to 0.3)	0.8
Avoid having surgery	3.7 (3.3–4.1)	3.1 (2.7–3.5)	0.6 (0.0 to 1.1)	0.5
*Which treatment do you prefer for your hip/knee OA?*, MR in % (CI)				
Replacement surgery	65 (57–74)	76 (69–83)	0.9 (0.7 to 1.0)	0.03
Non-surgical treatment options	20 (14–27)	10 (6–15)	1.9 (0.9 to 3.0)	0.07
I am not sure	15 (10–20)	13 (9–18)	1.1 (0.6 to 1.7)	0.7
Treatment scheduled at baseline, MR in % (CI)				
Surgery	65 (53–78)	87 (79–94)	0.8 (0.6 to 0.9)	0.002
Non-surgical treatment	35 (22–47)	13 (06–21)	2.6 (0.9 to 4.2)	0.07
Treatment received at 6 months follow-up, MR in % (CI)				
Surgery	79 (69–88)	88 (82–95)	0.9 (0.8 to 1.0)	0.08
Non-surgical treatment	21 (12–31)	12 (5–18)	1.8 (0.6 to 3.1)	0.2
HK-DQI, Knowledge score, MR in % (CI)				
Well informed > 60%	58 (50–66)	58 (50–66)	0.9 (0.8 to 1.2)	0.9
20%	20 (15–25)	18 (13–23)	1.1 (0.7 to 1.5)	0.6
60%	43 (37–49)	50 (44–55)	0.9 (0.7 to –1.0)	0.06
100%	38 (32–43)	32 (27–37)	1.2 (0.9 to –1.4)	0.2
Concordance, MR in % (CI)				
Received preferred treatment (e.g., surgical or non-surgical)	71 (63–78)	74 (67–81)	0.9 (0.8 to 1.0)	0.5
Preferred surgery and received surgery	60 (50–69)	72 (64–80)	0.8 (0.7 to 1.0)	0.04
Preferred non-surgical treatment and received non-surgical treatment	11 (5–18)	2 (0–5)	4.4 (–0.3 to 9.1)	0.1
No concordance, MR in % (CI)				
Did not receive preferred treatment (e.g., surgical or non-surgical)	29 (22–37)	26 (19–33)	1.1 (0.7 to 1.5)	0.5
Unsure about the preferred treatment	15 (10–20)	13 (8–18)	1.2 (0.6 to 1.7)	0.6
Preferred surgery and received non-surgical treatment	5 (2–9)	5 (2–8)	1.1 (0.1 to 2.1)	0.8
Preferred non-surgical treatment and received surgery	9 (5–12)	7 (5–10)	1.2 (0.5 to 1.8)	0.5

CI: 95% confidence interval; HK-DQI: Hip/Knee Osteoarthritis Decision Quality Instrument; MM: marginal mean; MMD: marginal mean difference; MR: marginal risk; MRR: marginal relative risk, SDM: shared decision-making.

Treatment received: 6 months post-enrolment; Concordance: alignment between the patient’s treatment preference and the treatment received; IPC: Informed Patient-Centered score if positive concordance score and a knowledge score of > 60%.

### Secondary outcomes

Patient-experienced engagement, measured by the CollaboRATE top score, was achieved by 94 patients (35%) in the intervention group and 102 patients (32%) in the control group. The MR was 35% (CI 27–42) in the intervention group and 32% (CI 25–39) in the control group. The MRR of achieving the top score was 1.1 (CI 0.8–1.4; P = 0.6).

Patients’ experiences of involvement in shared decision-making, assessed using the Hip/Knee OA Decision Quality Instrument (HK-DQI) SDM process score, were similar between groups, with an MD of 3.3 points (CI −1.5 to 8.0; P = 0.2).

Consultation time was longer in the intervention group, with an MD of 3.9 minutes compared with the control group (CI 0.1–7.7; P = 0.04).

Mean consultation time was consistently longer in the intervention group than in the control group across consecutive blocks of enrolled patients, with mean consultation times of approximately 29–32 minutes in the intervention group and 25–27 minutes in the control group. No clear change in consultation time was observed with increasing numbers of consultations in either group (Figure 2).

### Exploratory results

In the HK-DQI section “*What matters most to you?*”, MM were similar between groups for relieving pain and not being limited. For avoiding surgery, the intervention group reported a higher mean score than the control group, with an MMD of 0.6 points (CI 0.0–1.1; P = 0.08).

Regarding treatment preference at baseline, a higher proportion of patients in the control group preferred replacement surgery compared with the intervention group (76% vs 65%; P = 0.03), whereas preference for non-surgical treatment was more common in the intervention group (20% vs 10%; P = 0.07).

At baseline, fewer patients in the intervention group were scheduled for surgery compared with the control group 65% vs 87% with RR 0.8 (CI 0.6–0.9; P = 0.003). At 6 months’ follow-up, surgery remained more frequent in the control group (88% vs 79%), although the MRR was not statistically significant with RR 0.9 (CI 0.8–1.0; P = 0.08).

Knowledge scores were similar between groups. Overall, 58% of patients in both groups were categorized as well informed (> 60%).

Concordance between preferred and received treatment was comparable between groups. However, among patients who preferred surgery, receipt of surgical treatment was less frequent in the intervention group than in the control group 60% vs 72% with RR 0.8 (CI 0.7–0.9; P = 0.04).

## Discussion

This multicenter C-RCT examined whether the use of in-consult patient decision aids influenced decision quality and patient-experienced engagement or involvement among patients with severe hip or knee OA referred to orthopedic outpatient clinics. The groups showed similar estimates for decision quality and engagement and involvement, whereas consultation time was longer when in-consult patient decision aids were used. As no pre-specified and clinically validated MID are currently available for HK-DQI, IPC, or CollaboRATE, the observed CI cannot be interpreted in relation to the exclusion of clinically meaningful effects. Accordingly, the findings should be interpreted as inconclusive rather than as evidence of equivalence or absence of effect.

Knowledge scores were similar between groups, with 58% of patients categorized as well informed in both the intervention and control groups. Accordingly, no clear differences in decisional quality were observed. These findings contrast with earlier studies. Stacey et al. (2014) reported higher mean knowledge scores in the intervention group compared with usual care (71% vs 47%) in a Canadian trial of 142 OA patients considering TKA [19]. Further, Sepucha et al. (2017) observed mean knowledge scores of 41 (SD 27) and 40 (SD 28) in intervention and control groups, respectively, in a US cohort of 926 patients with either lumbar spinal stenosis or OA in the hip or knee, exposed to patient decision aids or usual care [20]. Differences in healthcare system organization may partly explain these discrepancies. In Denmark, patients typically undergo extended treatment with GPs and physiotherapists before being referred for treatment at hospitals, which contrasts with insurance-driven systems in the United States and Canada, where direct access to specialists allows earlier consultations before non-surgical options are fully explored. Consequently, Danish patients may have higher baseline knowledge and well-established treatment preferences at the time of hospital referral, reducing the patient decision aids’ relative impact on decision quality.

Comparing usual consultation with SDM with a patient decision aid for hip or knee OA also reports lower rates of high decision quality in control groups (25%–32%) than those observed in our study [20]. Additionally, a systematic review in 2021 highlighted that patient decision aids significantly improve decision quality. However, the certainty of evidence in that study, as assessed using the GRADE approach, was low to very low regarding patient decision aids’ ability to enhance decision quality and knowledge scores [21].

The lack of effect observed for the patient decision aids in this study may be explained by the timing of their delivery. Unlike many patient decision aids, which are typically provided prior to consultations, the in-consult patient decision aids method was introduced during the orthopedic consultation in accordance with the Danish implementation strategy [22]. While this approach enabled real-time discussion with surgeons, it may have limited patients’ opportunity to process and reflect on the information.

In addition, the high baseline knowledge and preparedness of the study population may have reduced the potential impact of the patient decision aids. Many patients had already undergone non-surgical treatments and were referred with established preferences, reduced quality of life, and a desire for surgery [23], which may have limited the influence of the patient decision aids during the consultation.

Patient-experienced engagement, assessed using CollaboRATE, showed similar proportions of top scores between groups, with 32% in the control group and 35% in the intervention group achieving the highest score. Although these proportions may appear modest, they are consistent with previously reported variation, ranging from 40–49% across medical specialties and 29–33% in orthopedic surgical settings [17]. Despite this, most patients received treatments concordant with their preferences (71% in the intervention group and 74% in the control group). A small proportion who initially preferred non-surgical treatment ultimately underwent surgery (9% and 7%, respectively), suggesting either a shift in preferences after the consultation or residual uncertainty during decision-making.

Fewer patients in the intervention group were scheduled for surgery compared with the control group (65% vs 87%), with rates increasing to 79% and 88%, respectively, at 6 months. This trend towards lower surgical rates in the intervention group has been observed with varying consistency in previous studies [21], although a similar tendency is reported by Sepucha et al. (2017) [20]. While these differences may not directly reflect the impact of the patient decision aids, they align with findings from other fields, such as back surgery and hysterectomy, where providing alternatives and allowing time for deliberation have been associated with reduced surgical rates [4].

Consultation length in this study reflects the Danish organization of orthopedic outpatient care, where about 30 minutes are allocated for the first consultation. In other countries, where the duration of consultations is considerably shorter, the implementation of in-consult patient decision aids may be even more challenging, thereby limiting the generalizability of our findings. Furthermore, health literacy is known to vary across Europe, with generally lower levels reported in Southern, Western, and Eastern countries compared with Northern Europe [24]. Such differences should be considered when interpreting our results, as they may influence the extent to which patient decision aids can support decision-making in various healthcare contexts.

### Limitations

First, the pragmatic design and inclusion across 2 departments posed a risk of selection bias, as surgeons were responsible for enrolment. Staff turnover, increased workloads, and efforts to reduce waiting times may have led to selective inclusion. Second, the lack of standardized criteria for joint replacement eligibility may have influenced participant selection and limits generalizability. Third, implementing SDM with a patient decision aid is challenging due to variability in clinicians’ attitudes, clinic workflows, and patients’ receptiveness. Surgeons in the intervention group reported difficulties with the paper-based patient decision aid, noting that some patients struggled to understand its purpose, while others arrived with pre-determined preferences. Feedback sessions were conducted to address these barriers and improve integration. Fourth, awareness of the study may have influenced control group surgeons to enhance patient information and involvement. Protocol adherence was monitored through unannounced observations, and surgeon-specific effects were accounted for in statistical analyses. Finally, the primary outcome, decision quality, is a complex measure based on knowledge scores regarding joint replacement outcomes. Including both surgical and non-surgical patients risked unequal access to joint replacement information, potentially influencing results. However, surgeons were instructed to deliver standardized joint replacement information to all patients during consultations.

### Conclusion

We showed similar estimates for decision quality and patient-experienced engagement and involvement between patients receiving an in-consult patient decision aid and those receiving usual consultation, while consultation time was longer in the intervention group.

## Notes

TAP, MLL, CMJ, KDS, and CV designed the study and contributed to the development of the statistical analysis plan. TAP conducted the study and served as project manager throughout. MLL and CV supported project management at their respective institutions. The final results were discussed, and the presentation of findings structured by TAP, MLL, CMJ, KDS, and CV. TAP prepared the initial draft of the manuscript, with MLL, CMJ, KDS, and CV providing revisions and approving the final version.

The authors would like to thank the orthopedic surgeons, project nurses, and outpatient clinic nurses at the Departments of Orthopedic Surgery at Lillebaelt University Hospital, Vejle, and Odense University Hospital, Odense and Svendborg, for their roles in the interventions, patient enrolment, and data management. They are grateful to Simon Kornvig for statistical support, and to OPEN for assistance with data management, REDCap, and OPEN storage. Finally, they sincerely thank all participating patients for their time and contribution.

Handling co-editors: Taco Gosens and Robin Christensen

*Acta* thanks Vesna Levašič, Maziar Mohaddes, and Walter van der Weegen for help with peer review of this manuscript.

## References

[1] Okafor L, Chen AF. Patient satisfaction and total hip arthroplasty: a review. Arthroplasty 2019; 1: 6. doi: 10.1186/s42836-019-0007-3. 35240763 PMC8787874

[2] Laigaard J, Aljuboori S M, Nikolajsen L, Mathiesen O, Lunn T H, Lindberg-Larsen M, et al. Chronic pain after primary total and medial unicompartmental knee arthroplasty for osteoarthritis: a Danish nationwide cross-sectional survey. Acta Orthop 2025; 96: 814-21. doi: 10.2340/17453674.2025.44898. 41189424 PMC12559960

[3] Elwyn G, Cochran N, Pignone M. Shared decision making—the importance of diagnosing preferences. JAMA Intern Med 2017; 177: 1239-40. doi: 10.1001/jamainternmed.2017.1923 28692733

[4] Stacey D, Légaré F, Lewis K, Barry M J, Bennett C L, Eden K B, et al. Decision aids for people facing health treatment or screening decisions. Cochrane Database Syst Rev 2017; 4: CD001431. doi: 10.1002/14651858.cd001431.pub5 28402085 PMC6478132

[5] Sepucha K R, Vo H, Chang Y, Dorrwachter J M, Dwyer M, Freiberg A A, et al. Shared decision-making is associated with better outcomes in patients with knee but not hip osteoarthritis: the DECIDE-OA randomized study. J Bone Joint Surg Am 2022; 104: 62-9. doi: 10.2106/jbjs.21.00064. 34437308

[6] Campbell M K, Piaggio G, Elbourne D R, Altman D G. Consort 2010 statement: extension to cluster randomised trials. BMJ 2012; 345: e5661. doi: 10.1136/bmj.e5661. 22951546

[7] Sepucha K R, Abhyankar P, Hoffman A S, Bekker H L, LeBlanc A, Levin C A, et al. Standards for UNiversal reporting of patient decision aid evaluation studies: the development of SUNDAE Checklist. BMJ Qual Saf 2018; 27(5): 380. doi: 10.1136/bmjqs-2017-006986. PMC5965362 29269567

[8] Pedersen T A, Lindberg-Larsen M, Jensen C M, Timm S, Steffensen K D, Varnum C. Impact of an in-consult patient decision aid on decisional quality, involvement, and health outcome for patients with severe hip or knee osteoarthritis: a study protocol for a multicentre, cluster randomised controlled trial (PATI-study). BMC Musculoskelet Disord 2025; 26: 584. doi: 10.1186/s12891-025-08856-w. 40597226 PMC12220521

[9] Stacey D, Legare F, Lyddiatt A, Giguere A M, Yoganathan M, Saarimaki A, et al. Translating evidence to facilitate shared decision making: development and usability of a consult decision aid prototype. Patient 2016; 9: 571-82. doi: 10.1007/s40271-016-0177-9. 27167076 PMC5107194

[10] Bennett C, Graham I D, Kristjansson E, Kearing S A, Clay K F, O’Connor A M. Validation of a preparation for decision making scale. Patient Educ Couns 2010; 78: 130-3. doi: 10.1016/j.pec.2009.05.012. 19560303

[11] Elwyn G, Durand M A, Song J, Aarts J, Barr P J, Berger Z, et al. A three-talk model for shared decision making: multistage consultation process. BMJ 2017; 359: j4891. doi: 10.1136/bmj.j4891. 29109079 PMC5683042

[12] Sepucha K. Hip and knee osteoarthritis: informed, patient-centered decision measure. Users guide; 2019. Available from: https://mghdecisionsciences.org/wp-content/uploads/2020/01/2019-hip-and-knee-ipc-user-guide.pdf.

[13] Sepucha K, Stacey D, Clay C, Chang Y, Cosenza C, Dervin G, et al. Decision quality instrument for treatment of hip and knee osteoarthritis: A psychometric evaluation. BMC Musculoskelet Disord 2011; 12: 149. doi: 10.1186/1471-2474-12-149 21729315 PMC3146909

[14] Elwyn G. CollaboRATE measure; 2013. Available from: http://www.glynelwyn.com/collaborate-measure.html.

[15] Barr P J, Thompson R, Walsh T, Grande S W, Ozanne E M, Elwyn G. The psychometric properties of CollaboRATE: a fast and frugal patient-reported measure of the shared decision-making process. J Med Internet Res 2014; 16: e2. doi: 10.2196/jmir.3085. 24389354 PMC3906697

[16] Bilbao A, García-Pérez L, Arenaza J C, García I, Ariza-Cardiel G, Trujillo-Martín E, et al. Psychometric properties of the EQ-5D-5L in patients with hip or knee osteoarthritis: reliability, validity and responsiveness. Qual Life Res 2018; 27: 2897-908. doi: 10.1007/s11136-018-1929-x. 29978346

[17] Brodney S, Fowler F J Jr, Barry M J, Chang Y, Sepucha K. Comparison of three measures of shared decision making: SDM Process_4, CollaboRATE, and SURE scales. Med Decis Mak 2019; 39: 673-80. doi: 10.1177/0272989X19855951 PMC6791732 31226911

[18] McBee M. Modeling outcomes with floor or ceiling effects: an introduction to the Tobit Model. Gifted Child Q 2010; 54: 314-20. doi: 10.1177/0016986210379095.

[19] Stacey D, Hawker G, Dervin G, Tugwell P, Boland L, Pomey M-P, et al. Decision aid for patients considering total knee arthroplasty with preference report for surgeons: a pilot randomized controlled trial. BMC Musculoskelet Disord 2014; 15: 54. doi: 10.1186/1471-2474-15-54. 24564877 PMC3937455

[20] Sepucha K, Atlas S J, Chang Y, Dorrwachter J, Freiberg A, Mangla M, et al. Patient decision aids improve decision quality and patient experience and reduce surgical rates in routine orthopaedic care: a prospective cohort study. J Bone Joint Surg 2017; 99: 1253-60. doi: 10.2106/jbjs.16.01045 28763411

[21] Pacheco-Brousseau L, Charette M, Poitras S, Stacey D. Effectiveness of patient decision aids for total hip and knee arthroplasty decision-making: a systematic review. Osteoarthritis Cartilage 2021; 29: 1399-411. doi: 10.1016/j.joca.2021.07.006. 34302958

[22] Olling K, Bechmann T, Madsen P H, Jakobsen E H, Toftdahl D B, Hilberg O, et al. Development of a patient decision aid template for use in different clinical settings. Eur J Pers Cent Healthc 2019; 7.

[23] Moseng T, Vliet Vlieland T P M, Battista S, Beckwée D, Boyadzhieva V, Conaghan P G, et al. EULAR recommendations for the non-pharmacological core management of hip and knee osteoarthritis: 2023 update. Ann Rheum Dis 2024; 730-40. doi: 10.1136/ard-2023-225041. 38212040 PMC11103326

[24] Baccolini V, Rosso A, Di Paolo C, Isonne C, Salerno C, Migliara G, et al. What is the prevalence of low health literacy in European Union member states? A systematic review and meta-analysis. J Gen Intern Med 2021; 36: 753-61. doi: 10.1007/s11606-020-06407-8. 33403622 PMC7947142

